# Non‐small cell lung cancer with tumor proportion score > 90% could increase the risk of severe immune‐related adverse events in first‐line treatments with immune checkpoint inhibitors: A retrospective single‐center study

**DOI:** 10.1111/1759-7714.14576

**Published:** 2022-07-12

**Authors:** Yuki Akazawa, Aki Yoshikawa, Masaki Kanazu, Yukihiro Yano, Toshihiko Yamaguchi, Masahide Mori

**Affiliations:** ^1^ Department of Thoracic Oncology National Hospital Organization Osaka Toneyama Medical Center Toyonaka City Japan

**Keywords:** adenocarcinoma, immune checkpoint inhibitor, immune‐related adverse event, non‐small cell lung cancer

## Abstract

**Background:**

Since 2015, immune checkpoint inhibitors have been a clinical treatment strategy for patients with advanced or recurrent non‐small cell lung cancer (NSCLC). However, the relationship between immune‐related adverse event (irAE) risk factors and patient clinical characteristics is unclear. This study aimed to evaluate the relationship between irAE risk and the clinical characteristics of patients with NSCLC.

**Methods:**

We included patients with advanced or recurrent NSCLC with known programmed death‐ligand 1 expression levels treated with immune checkpoint inhibitors. We retrospectively examined the medical records of 260 patients with NSCLC (March 2016–November 2020) and analyzed the relationship between the patient clinical characteristics and irAEs.

**Results:**

Our retrospective analysis revealed that tumor proportion score (TPS) ≥ 90% and adenocarcinoma histology were independent risk factors for irAEs (odds ratio: 3.750 95% confidence interval [CI]: 1.58–8.89 and 0.424 95% CI: 0.19–0.97, respectively) in first‐line treatment. However, in patients receiving second‐ or later‐line treatments, no clinical characteristics were identified as risk factors for irAEs. Furthermore, no difference was observed in the response rates to first‐line treatments between the TPS ≥ 90% and TPS < 90% groups (74% vs. 71%, *p* = 0.83). In later‐line treatments, the TPS ≥ 90% group had a better response rate than the TPS < 90% group (55% vs. 17%, *p* < 0.05). However, no significant differences in overall survival were observed in either of the groups.

**Conclusions:**

TPS ≥ 90% and adenocarcinoma histology were independent risk factors for irAEs in previously untreated patients with advanced or recurrent NSCLC. Therefore, patients at high risk of irAEs require additional monitoring.

## Graphical abstract

Patients with TPS ≥ 90% has significantly higher possibility of developing severe immune‐related adverse events than patients with TPS < 90%.

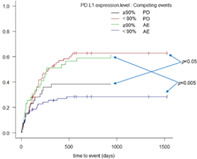

## INTRODUCTION

Lung cancer is the leading cause of cancer‐related mortality worldwide.^1^ Immune checkpoint inhibitors (ICIs) have recently been introduced as a clinical treatment strategy for advanced or recurrent non‐small cell lung cancer (NSCLC) and extensive‐disease small cell lung cancer. Five types of ICIs are currently available for treating NSCLC. In phase 3 clinical trials worldwide, both ICI monotherapy^2^, ^3^, ^4^, ^5^, ^6^ and ICI combination therapy^7^, ^8^, ^9^, ^10^, ^11^ showed better responses and survival benefits than conventional chemotherapy treatments.

The American Society of Clinical Oncology (ASCO) guidelines^12^ for stage IV NSCLC without driver alterations strongly recommend single‐agent pembrolizumab as a first‐line treatment for patients with ≥50% expression of programmed death‐ligand 1 (PD‐L1). Additional treatment options include ICI and chemotherapy combination regimens. Contrastingly, combination therapy with ICIs and cytotoxic chemotherapy agents is recommended for NSCLC with low (1%–49%) or negative (0%) PD‐L1 expression. Pembrolizumab monotherapy may be a treatment option for NSCLC in selected cases with low PD‐L1 expression.^4^ Most major clinical guidelines for advanced or recurrent NSCLC follow treatment strategies similar to the ASCO guidelines.^13^, ^14^

Most clinical trials report that approximately 10% of patients in ICI treatment groups develop severe immune‐related adverse events (irAEs). Regarding first‐line clinical trials, the KEYNOTE‐024 study reported an incidence of 9.7% of irAEs with a score ≥3, according to the Common Terminology Criteria for Adverse Events (CTCAE), in the pembrolizumab monotherapy group.^3^ The KEYNOTE‐189^7^ and KEYNOTE‐407^8^ studies reported severe irAE rates of 8.9% and 10.8% in the pembrolizumab and chemotherapy combination groups, respectively. Pooled analysis of nivolumab for previously treated patients with NSCLC yielded an incidence of 5% of irAEs with a score ≥3.^5^ The KEYNOTE‐010 study,^2^ primarily aiming to reveal the overall survival and progression‐free survival rates of pembrolizumab monotherapy for previously treated patients with NSCLC with PD‐L1 expression ≥1%, reported a rate of 10.4% for severe adverse events. OAK, a phase 3 trial that compared atezolizumab with docetaxel in previously treated patients with NSCLC, reported an incidence of 15% for treatment‐related adverse events of grade ≥3.^6^ However, the association between patient clinical characteristics and development of severe irAEs remains unknown. Risk factors for irAEs are not mentioned in any of the large‐scale phase 3 clinical trials listed above. Thus, this study aimed to assess the relationship between the risk of irAEs and patient clinical characteristics.

## METHODS

### Study design

This retrospective analysis was conducted at the Department of Thoracic Oncology, National Hospital Organization Osaka Toneyama Medical Center, Osaka, Japan, in accordance with the Declaration of Helsinki and Recommendations for the Conduct, Reporting, Editing and Publication of Scholarly Work in Medical Journals. The study protocol was approved by the Institutional Review Board of Osaka Toneyama Medical Center (approval no.: TNH‐P‐2021021). This study did not require patients to provide informed consent because all data were retrospectively and anonymously collected. This analysis aimed to identify clinical factors associated with the onset of severe irAEs. We retrospectively examined all patients with advanced or recurrent NSCLC, with a known tumor proportion score (TPS) of the cancer pathological tissue, who started ICI monotherapy or combination regimens at Osaka Toneyama Medical Center between March 2016 and November 2020. More than 350 patients with NSCLC were treated with ICIs during the study period. We selected 260 patients with a known TPS because we aimed to assess the relationship between the risk of irAEs and TPS. Patients without known TPS were excluded from the study. Tumor specimens from formalin‐fixed paraffin‐embedded samples were stained with PD‐L1 IHC 22C3 pharmDx assay (Dako, Agilent Technologies), the only assay approved by the health insurance system in Japan.

The treatment strategy for stage II–IIIB NSCLC differs from that for stage IIIC–IV in terms of maintenance therapy with durvalumab. Therefore, we excluded patients with stage II–IIIB NSCLC treated with durvalumab following curative chemoradiotherapy. We defined severe irAEs as follows: interstitial lung disease (ILD) of any grade and other irAEs of grades 2–5 according to the CTCAE (version 4.0), requiring temporary or permanent treatment discontinuation or intervention with steroids.

### Data collection and statistical analysis

Of the 260 retrospectively analyzed patients, 114 (44%) patients were included in the first‐line treatment group, and 146 (56%) patients were included in the second‐ or later‐line treatment group. The following data were collected: (i) patient characteristics (sex, age [divided into the following age groups: ≤59, 60–69, 70–79, and ≥ 80 years], smoking status, Eastern Cooperative Oncology Group performance status [ECOG‐PS], a history of other malignancies, histology or cytology test results [adenocarcinoma or nonadenocarcinoma], driver status, PD‐L1 expression level [negative, <1%; low, 1–49%; high, ≥50%], and TPS [≥90% or<90%]); (ii) treatment characteristics (type of treatment administered [first‐line or second‐ or later‐line], type of treatment regimen [monotherapy or combination regimen], and type of ICI administered [nivolumab, pembrolizumab, or atezolizumab]); and (iii) safety and efficacy (type of irAE, time to the onset of irAEs, time to treatment failure, and response and survival time). We applied TPS ≥ 90% as an examining parameter as it is a prognostic factor for better response and longer survival than TPS < 90%.^15^, ^16^

Each investigator evaluated the treatment response and adverse events using both the new Response Evaluation Criteria in Solid Tumors (RECIST): revised RECIST guideline (version 1.1)^17^ and the CTCAE (version 5.0).^18^ The overall response rate was defined as the percentage of patients achieving either complete or partial response to treatment. Overall survival was defined as the interval (in days) from the first ICI administration until death or the date of last follow‐up, and time‐to‐event as the interval (in days) from the first ICI administration until the date of onset of irAEs or disease progression (whichever occurred first). Patients who were lost to follow‐up were censored at the last date of contact. All statistical analyses were conducted using EZR (version 2.4–0; Saitama Medical center, Jichi Medical University, Saitama, Japan).^19^, ^20^ Significant difference was defined as a *p*‐value <0.05. Median overall survival and median time‐to‐event were calculated using the Kaplan–Meier method, and treatments were compared using the log‐rank test. Multivariate analysis of risk factors for irAEs, including the year of diagnosis, sex, age, smoking history, history of other malignancies, TPS, histological diagnosis, driver status, ICI regimen, PD‐L1 expression level, and main ICI, was performed using a logistic regression model.

All patients with epidermal growth factor receptor (EGFR) mutations received ICIs after treatment failure with EGFR‐tyrosine kinase inhibitors (EGFR‐TKIs). In these cases, we excluded EGFR‐TKI treatment from the treatment lines, and treatment with ICI‐chemotherapy was considered the first‐line treatment.

## RESULTS

### Patient clinical characteristics

The patient clinical characteristics are shown in Table 1. We included 260 patients in the analysis: 114 (44%) were in the first‐line treatment group; 184 (71%) were men (90 [79%] in the first‐line treatment group and 94 [65%] in the second‐ or later‐line treatment group; *p* < 0.05); the median age was 70.7 years (95% confidence interval [CI]: 69.7–71.8 years; 71.7 [70.1–73.3] years in the first‐line treatment group and 70.0 [68.6–71.4] years in the second‐ or later‐line treatment group; *p* = 0.84); 202 (78%) patients had a good level of functioning, with an ECOG‐PS score of 0–1 (95 [83%] in the first‐line treatment group and 107 [73%] in the second‐ or later‐line treatment group; *p* = 0.07); and 210 (81%) patients were negative for driver mutations (100 [88%] in the first‐line treatment group and 110 [75%] in the second‐ or later‐line treatment group; *p* = 0.38). A total of 230 (88%) patients had a history of smoking (105 [92%] in the first‐line treatment group and 125 [73%] in the second‐ or later‐line treatment group; *p* = 0.12). Twenty‐three (9%) patients had a history of other malignancies (16 [14%] in the first‐line treatment group and seven [4%] in the second‐ or later‐line treatment group; *p* < 0.05). Seven patients had slight ILD as a pre‐existing condition without any symptoms (two [1.8%] in the first‐line treatment group and five [3%] in the second‐ or later‐line treatment group). Fifty‐one (45%) patients in the first‐line treatment group received ICI combination regimens, and 146 (100%) in the second‐ or later‐line treatment group received ICI monotherapy. Nine patients in the first‐line treatment group and 31 in the second‐ or later‐line treatment group received EGFR‐TKIs prior to the ICI regimen, and none of them developed ILD induced by EGFR‐TKIs. Details of the previous EGFR‐TKI treatments received by patients are presented in Table 2. Two patients in the first‐line treatment group and 16 in the second‐ or later‐line treatment group previously received at least two regimens of EGF‐TKI treatments, and none of them developed ILD prior to ICI treatment.

**TABLE 1:** Comparison of patient clinical characteristics in the first‐line and second‐ or later‐line treatment groups

Clinical characteristics		First‐line treatment, *n* = 114	Second‐ or later‐line treatment, *n* = 146	*p*‐value
Sex	Male	90 (79%)	94 (65%)	<0.05
	Female	24 (21%)	52 (35%)	
Median age (years) (95% CI)		71.7 (70.1–73.3)	70.0 (68.6–71.4)	0.84
Age group (years)	≤59	8 (7%)	17 (12%)	0.055
	60–69	30 (34%)	49 (34%)	
	70–79	54 (47%)	66 (45%)	
	≥80	22 (19%)	13 (9%)	
ECOG‐PS	Good (0–1)	95 (83%)	107 (73%)	0.07
	Poor (2–4)	19 (17%)	39 (17%)	
Smoking status	Current/former	105 (92%)	125 (86%)	0.12
	Never	9(8%)	21 (14%)	
History of malignancy	Yes	16 (14%)	7 (4%)	<0.05
	No	98 (86%)	139 (95%)	
Histology	Adenocarcinoma	60 (53%)	85 (58%)	0.38
	Nonadenocarcinoma	54 (47%)	61 (42%)	
Driver status	Negative	100 (88%)	110 (75%)	<0.05
	Positive	14 (12%)	36 (25%)	
	(EGFR/KRAS/others)	(9/4/1)	(31/1/4)	
Regimen	Combination	51 (45%)	0 (0%)	
	Monotherapy	63 (55%)	146 (100%)	
PD‐L1 expression	Negative	5 (4%)	50 (34%)	<0.001
	Low	26 (23%)	61 (42%)	
	High	83 (73%)	35 (24%)	
TPS ≥ 90%		39 (34%)	18 (12%)	<0.001
ICI	Nivolumab	0	63 (43%)	
	Pembrolizumab	98 (86%)	57 (39%)	
	Atezolizumab	16 (14%)	26 (18%)	

Abbreviations: CI, confidence interval; ECOG‐PS, Eastern Cooperative Oncology Group performance status; EGFR, epidermal growth factor receptor; ICI, immune checkpoint inhibitor; KRAS, Kirsten rat sarcoma viral oncogene; PD‐L1, programmed death‐ligand 1; TPS, tumor proportion score.

**TABLE 2:** Details of treatment regimens

Group	No. of patients
First‐line treatment group	
Pembrolizumab	63
Carboplatin/nab‐paclitaxel/pembrolizumab	23
Carboplatin/paclitaxel/bevacizumab/atezolizumab	3
Carboplatin/nab‐paclitaxel/atezolizumab	3
Cisplatin/pemetrexed/pembrolizumab	2
Carboplatin/pemetrexed/pembrolizumab	10
Carboplatin/pemetrexed/atezolizumab	6
Carboplatin/pemetrexed/atezolizumab/bevacizumab	4
Previous treatment with EGFR‐TKI: Gefitinib	2
Erlotinib	3
Afatinib	5
Osimertinib	2
Second‐ or later‐line treatment group	
Nivolumab	63
Pembrolizumab	57
Atezolizumab	26
Previous treatment with EGFR‐TKI: Gefitinib	18
Erlotinib	9
Afatinib	14
Osimertinib	16

Abbreviation: EGFR‐TKI, epidermal growth factor receptor‐tyrosine kinase inhibitor.

Overall, 55 (21%), 87 (34%), and 118 (45%) patients had negative, low, and high levels of PD‐L1 expression, respectively. For each PD‐L1 expression group, five (4%), 26 (23%), and 83 (73%) patients, respectively, were in the first‐line treatment group, and 50 (34%), 61 (42%), and 35 (24%), respectively, were in the second‐ or later‐line treatment group (*p* < 0.001). Among all patients, 57 (22%) had TPS ≥ 90% (39 in the first‐line treatment group and 18 in the second‐ or later‐line treatment group).

The treatment regimens were as follows (Table 2). In the first‐line treatment group, 63 (55%) patients received pembrolizumab monotherapy.^3^, ^4^ A total of 23 patients with squamous NSCLC received carboplatin/nab‐paclitaxel/pembrolizumab,^8^ and 28 with nonsquamous‐squamous NSCLC received other ICI combination regimens (three received carboplatin/paclitaxel/bevacizumab/atezolizumab,^9^ three received carboplatin/nab‐paclitaxel/atezolizumab,^11^ 12 received cisplatin or carboplatin/pemetrexed/pembrolizumab,^7^ six received carboplatin/pemetrexed/atezolizumab,^10^ and four received carboplatin/pemetrexed/atezolizumab/bevacizumab in a clinical trial^21^). In the second‐ or later‐line treatment group, 63, 57, and 26 patients received nivolumab,^5^ pembrolizumab,^2^ and atezolizumab^6^ monotherapy, respectively.

### Analysis of risk factors for severe irAEs

Table 3 shows the univariate analysis of severe irAEs according to the treatment line. ICI combination regimens were adapted only for patients receiving first‐line treatment, and nivolumab was approved for second‐ or later‐line monotherapy. The patient characteristics that differed between the two groups were examined separately. According to the frequency of severe irAEs, a univariate analysis of patient clinical characteristics in the first‐line treatment group showed that TPS ≥ 90% and adenocarcinoma histology were risk factors for severe irAEs. Multivariate analysis showed that both TPS ≥ 90% and adenocarcinoma histology were independent risk factors for severe irAEs in first‐line treatment (odds ratio for TPS ≥ 90%: 3.750 [95% CI: 1.58–8.89], *p* < 0.005; odds ratio for nonadenocarcinoma vs. adenocarcinoma: 2.24 [95% CI: 1.01–4.98], *p* < 0.05). However, no risk factors for severe irAEs were detected in the second‐ or later‐line treatment group.

**TABLE 3:** Analysis of irAE risk factors according to treatment lines

		Univariate analysis of first‐line treatment			Multivariate analysis of first‐line treatment		Univariate analysis of second‐ or later‐line treatment		
All	irAE	No	Yes	*p*‐value	Odds ratio	*p*‐value	No	Yes	*p*‐value
Sex	Male	48	42	0.26			79	15	1
	Female	16	8				44	8	
Age group (years)	≤59	6	2	0.07			14	3	0.36
	60–69	18	12				41	8	
	70–79	33	21				59	8	
	≥80	7	15				9	4	
Smoking history	Never	6	3	0.73			17	3	1
	Current/former	58	47				106	20	
History of other malignancy	No	57	41	0.29			117	22	1
	Yes	7	9				6	1	
ECOG‐PS	Good (0–1)	52	43	0.62			87	20	0.13
	Poor (2–4)	12	7				36	3	
Histology	Adenocarcinoma	28	32	<0.05	0.424	<0.05	74	11	0.36
	Nonadenocarcinoma	36	18		(0.19–0.97)		51	10	
Driver status	Negative	56	44	1			90	20	0.20
	Positive	8	6				33	3	
ICI regimen	Combination	29	22	1					
	Monotherapy	32	25						
PD‐L1 expression	Negative	2	3	0.69			42	8	0.11
	Low	16	10				55	6	
	High	46	37				26	9	
TPS ≥ 90%	No	51	24	<0.001	3.750	<0.005	107	21	0.74
	Yes	13	26		(1.58–8.89)		16	2	
ICI	Nivolumab	‐‐	‐‐	0.79			53	10	1
	Pembrolizumab	54	44				48	9	
	Atezolizumab	10	6				22	4	

Abbreviations: irAE, immune‐related adverse event; ECOG‐PS, Eastern Cooperative Oncology Group performance status; ICI, immune checkpoint inhibitor; PD‐L1, programmed death‐ligand 1; TPS, tumor proportion score.

### Details of irAEs

Table 4 shows the details of the severe irAEs according to the treatment line. The frequency of severe irAEs was higher in the first‐line than in the second‐ or later‐line treatment group. Fifty‐one (45%) patients in the first‐line treatment group and 23 (16%) patients in the second‐ or later‐line treatment group developed irAEs requiring treatment interruption or intervention with steroids. After focusing the analysis on TPS ≥ 90%, the risk of irAEs was higher in patients receiving first‐line treatment (26 patients [67%]) than in those receiving second‐ or later‐line treatment (two patients [11%]). ILD was the most frequently observed severe irAE in both groups (16 [14%] patients in the first‐line treatment group and eight [6%] patients in the second‐ or later‐line treatment group). One patient in the first‐line treatment group and one in the second‐ or later‐line treatment group, both with low TPS, died from ILD; those patients were refractory to high doses of steroids. Seven patients had a medical history of ILD, two of which received pembrolizumab monotherapy and atezolizumab combination regimens as first‐line treatment, and five of which received monotherapy (two received pembrolizumab; two received nivolumab; and one received atezolizumab). Among them, one patient with PD‐L1 expression levels of 95% developed grade 2 ILD 7 days after the first administration of nivolumab.

**TABLE 4:** Details of irAEs: Percentage of patients who developed irAEs according to treatment lines

(TPS ≥ 90%)	First‐line treatment, *n* = 114 (*n* = 39)	Second‐ or later‐line treatment, *n* = 146 (*n* = 18)
irAE, yes	50 (44%)	21 (14%)
(TPS ≥ 90%)	(26, 67%)	(2, 11%)
Skin	15 (9)	3 (0)
Gastrointestinal	10 (7)	3 (0)
Liver, cholecystitis	6 (3)	2 (0)
Endocrine	8 (3)	7 (1)
Pulmonary	16 (6)	7 (1)
Renal	4 (3)	0 (0)
Others	6 (3)	2 (0)
Grade 5	1, ILD	1, ILD
≥2 irAEs	17 (9)	3 (1)
Retreatment with ICIs	23 (16)	8 (1)

Abbreviations: ICI, immune checkpoint inhibitor; ILD, interstitial lung disease; irAE, immune‐related adverse event; TPS, tumor proportion score.

The numbers in parentheses represent the numbers of patients with a TPS ≥ 90% who developed irAEs.

The gastrointestinal irAE of diarrhea was observed in nine patients in the first‐line treatment group and in three patients in the second‐ or later‐line treatment group. Six patients in the first‐line treatment group and none in the second‐ or later‐line treatment group had TPS ≥ 90%. One patient in the first‐line treatment group with TPS ≥ 90% developed grade 3 diarrhea; the other 11 patients had grade 2 diarrhea. All patients responded to steroids.

Some patients developed ≥2 severe irAEs concurrently or sequentially (17 [15%] and three [2%] patients in the first‐line and second‐ or later‐line treatment groups, respectively). In cases with mild and manageable irAEs, treatment with ICIs was continued or restarted with medication and close observation for the recurrence of severe irAEs. Approximately half of the patients experiencing severe adverse events restarted ICI treatment. Seven patients in the first‐line treatment group and eight in the second‐ or later‐line treatment group developed irAEs, even after discontinuing ICIs, owing to disease progression.

### Time‐to‐event (irAEs or progressive disease)

**FIGURE 1:**
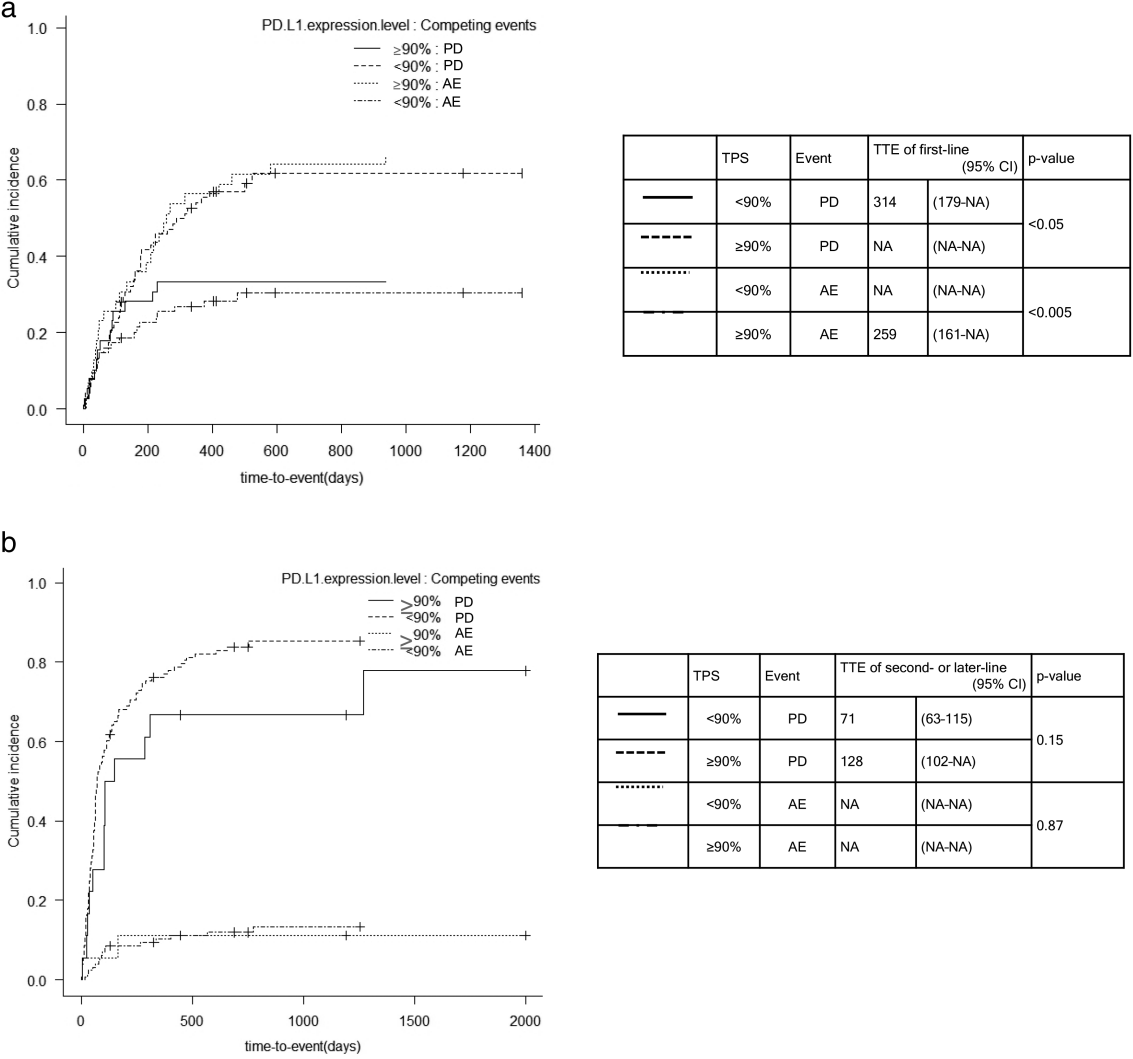
Cumulative incidence for assessing the risk for time‐to‐event. (a) Time‐to‐event in patients in the first‐line treatment group and (b) time‐to‐event in patients in the second‐ or later‐line treatment group. (c) In the first‐line treatment group, patients with TPS ≥ 90% had a statistically higher possibility of developing severe irAE. Conversely, no difference in the incidence of irAE were detected in the second or later‐line treatment group. Abbreviations: AE, adverse event; NA, not assessed; PD, programmed death; TPS, tumor proportion score; TTE, time‐to‐event

Figure 1 shows the cumulative incidence for assessing time‐to‐event (irAEs or progressive disease, whichever occurred first). In the first‐line treatment group, the incidence of severe irAEs was significantly higher, and disease progression was reduced in the TPS ≥ 90% group than in the TPS < 90% group (*p* < 0.005 and *p* < 0.05, respectively). In patients with TPS ≥ 90%, the median time to irAEs was 259 days (95% CI: 161–not assessed [NA]), and the median time to disease progression was not assessed (95% CI: NA–NA). Conversely, in the TPS < 90% group, the median time to irAEs was not assessed (95% CI: NA–NA), and the median time to disease progression was 314 days (95% CI: 179–NA). In the second‐ or later‐line treatment group, no difference was recorded in the risk of developing irAEs (*p* = 0.87) or disease progression (*p* = 0.15) between the TPS ≥ 90% and TPS < 90% groups. The median time to irAEs was not assessed in either group. The median time to disease progression was 128 days (95% CI: 102–NA) in the TPS ≥ 90% group and 71 days (95% CI: 63–115) in the TPS < 90% group.

No difference was observed in the risk of developing early irAEs between the TPS ≥ 90% and the TPS < 90% groups. Seven (18%) patients with TPS ≥ 90% and 11 (15%) with TPS < 90% in the first‐line treatment group, and one (5%) patient with TPS ≥ 90% and five (5%) with TPS < 90% in the second‐ or later‐line treatment group, developed severe irAEs within 60 days of the first ICI administration.

### Efficacy

**FIGURE 2:**
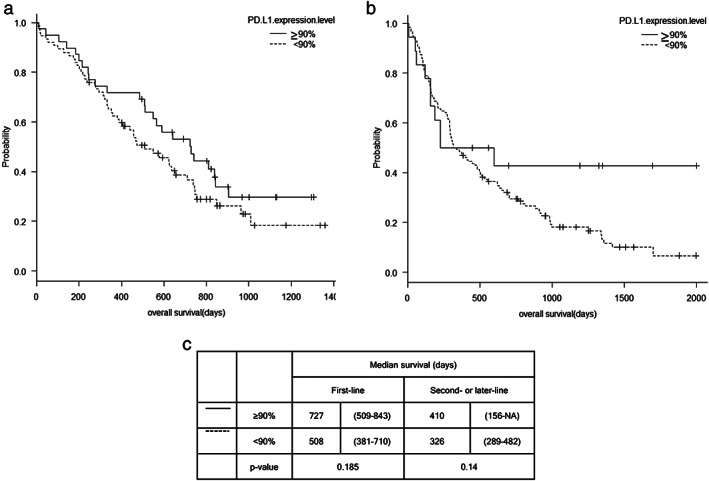
Overall survival according to ICI regimens. Kaplan–Meier survival curves and comparison of survival between TPS ≥ 90% group and TPS < 90% groups according to treatment lines. (a) Overall survival of the first‐line treatment group and (b) overall survival of the second‐ or later‐line treatment group. (c) No difference in overall survival time was observed between the TPS ≥ 90% group and TPS < 90% group in both first‐ and later‐line treatment groups. Abbreviations: ICI, immune checkpoint inhibitors; NA, not assessed; TPS, tumor proportion score

At the time of the data cutoff on December 28, 2021, 66 patients were alive, 192 death events were recorded, and two patients were lost to follow‐up. Two patients died from other malignancies (colon cancer and hepatocellular carcinoma). The response rate was 74% in the first‐line treatment group (complete remission, 12; partial response, 70) and 22% in the second‐ or later‐line treatment group (complete remission, three; partial response, 29). In patients receiving first‐line treatment, no difference was recorded in the response rates between the TPS ≥ 90% and TPS < 90% groups (74% vs. 64%; *p* = 0.298). However, in patients receiving second‐ or later‐line treatment, the TPS ≥ 90% group had a significantly better response rate than the TPS < 90% group (56% vs. 17%; *p* < 0.05). The median overall survival between the TPS ≥ 90% and TPS < 90% groups was not statistically different in either the first‐line treatment (727 days [95% CI: 509–843] vs. 508 days [95% CI: 381–710], *p* = 0.185) or the second‐ or later‐line treatment groups (410 days [95% CI: 156–NA] vs. 326 days [95% CI: 289–482], *p* = 0.14; Figure 2).

## DISCUSSION

This retrospective study identified TPS ≥ 90% and adenocarcinoma histology as risk factors for irAEs in previously untreated patients with NSCLC. However, we could not identify any risk factors for severe irAEs in the second‐ or later‐line treatment group. Several worldwide phase 3 clinical trials of first‐line treatments for advanced NSCLC have reported that the incidence of grade ≥3 irAEs was approximately 10%.^2^, ^3^, ^5^, ^6^, ^7^, ^8^ However, no trial has reported the risk factors for severe irAEs.

Our research primarily aimed to clarify the relationship between TPS and irAEs. Few studies have focused on predictive factors for severe irAEs. Fujii et al.^22^ retrospectively analyzed 290 patients with advanced solid cancer to determine the incidence of irAEs, risk factors, and their association with treatment outcomes. However, they could not determine risk factors while evaluating the biochemical analysis of blood samples.^22^ A retrospective clinical study of 44 patients by Sugisaka et al.,^20^ using univariate analysis, reported that high PD‐L1 expression, primary therapy, and ECOG‐PS 0 were independent risk factors for irAEs. Six (27.3%) patients in the first‐line treatment group had severe irAEs. A multicenter retrospective study of first‐line treatment with pembrolizumab monotherapy by Edahiro et al.^15^ showed no statistical difference in the incidence of irAEs between the TPS ≥ 90% and TPS 50%–89% groups. Furthermore, the two groups had similar response and disease control rates. However, after 120 days, patients with TPS ≥ 90% had greater survival benefits than those with TPS of 50%–89%.^14^ When these two investigations were planned, pembrolizumab was approved as monotherapy for previously untreated patients with NSCLC with high PD‐L1 expression and previously treated patients with NSCLC with TPS ≥1%.

In our retrospective analysis, TPS ≥ 90% was correlated with a significantly higher risk of irAEs in first‐line treatment. Numerous researchers have reported that patients with grade 3–4 irAEs, caused by nivolumab monotherapy as the second‐ or later‐line treatment, had better survival outcomes and responses than those without grade 3–4 irAEs.^23^, ^24^, ^25^, ^26^ For instance, Haratani et al.^24^ retrospectively analyzed 134 patients with NSCLC who received nivolumab as second‐ or later‐line treatment. Patients with irAEs showed statistically longer median progression‐free survival and median overall survival than those without irAEs.^24^ In contrast, a pooled analysis of three atezolizumab combination regimens (IMpower130,^11^ IMpower132,^10^ and IMpower150^9^) by Socinski et al.^27^ suggested that mild irAEs of grades 1–2 were associated with longer survival, and severe irAEs of grade 3–4 led to shorter survival owing to treatment interruption or discontinuation (ASCO 2021 #9002).^27^ These results suggest that treatment‐naïve patients have immune system differences compared with pretreated patients; hence, irAEs were frequently observed among patients receiving first‐line treatment.

During the treatment process, we should focus on treatment efficacy and safety. Few studies have examined the risk factors for irAEs. A retrospective study by Suresh et al.^28^ reported that squamous histological‐type tumors posed a significantly higher risk of ILD than other histological types. Conversely, we showed that adenocarcinoma histology was a risk factor for irAEs. To the best of our knowledge, no other study has reported the relationship between irAEs and specific histological types. Nevertheless, our results are controversial, and further research is warranted.

This study has some limitations. The clinical characteristics of patients were not well‐balanced between the TPS ≥ 90% and TPS < 90% groups and the first‐line and second‐ or later‐line treatment groups, owing to the retrospective nature of the study. Most patients without driver mutations were recently more likely to receive ICIs as first‐line treatment if they did not have contraindications for ICIs.^29^ We defined any grade of ILD as a severe irAE if it required treatment interruption or intervention with steroids. Our definition of irAE was wider than that in major clinical trials. Therefore, the number of reported irAEs in our analysis was higher than that in phase 3 clinical trials. Conducting a prospective clinical trial with a primary endpoint to assess the incidence and severity of irAEs is arduous because of their unexpected or accidental nature. The accumulation of real‐world data is warranted to clarify whether TPS ≥ 90% is related to severe irAEs and whether it contributes to better survival in patients who receive ICIs as a first‐line treatment alone. Another limitation of this study was its sample size. TPS ≥ 90% was identified as a risk factor for irAEs, particularly during first‐line treatment. However, our sample size was too limited to distinguish the outcome according to the severity of irAEs. Additionally, we could not determine the types of irAEs associated with better survival.

In conclusion, to the best of our knowledge, this is the first analysis of the relationship between patient clinical characteristics and the risk of irAEs. TPS ≥ 90% was strongly associated with the risk of developing irAEs only in patients receiving first‐line treatment with ICIs. TPS ≥ 90% and adenocarcinoma histology may be predictive factors for severe irAEs in previously untreated patients with NSCLC; hence, severe adverse events should be closely monitored. Further investigation of real‐world data is warranted to reveal the relationship between TPS and the risk for severe irAEs.

## CONFLICT OF INTEREST

Yuki Akazawa received research funding from Chugai Pharmaceutical (Tokyo, Japan). Masaki Kanazu received honoraria for lectures and presentations from AstraZeneca K.K. (Osaka, Japan), Ono Pharmaceutical (Osaka, Japan), Shionogi Pharmaceutical (Osaka, Japan), Chugai Pharmaceutical (Tokyo, Japan), MSD K.K (Tokyo, Japan), Boehringer Ingelheim (Ingelheim, Germany), and Eli Lilly Japan (Kobe, Japan). Yukihiro Yano received research funding from Chugai Pharmaceutical (Tokyo, Japan). Toshihiko Yamaguchi received research funding from Chugai Pharmaceutical (Tokyo, Japan). Masahide Mori received lecture fees from AstraZeneca K.K. (Osaka, Japan), MSD K.K (Tokyo, Japan), Ono Pharmaceutical (Osaka, Japan), Eli Lilly Japan (Kobe, Japan), Boehringer Ingelheim (Ingelheim, Germany), Novartis Japan (Tokyo, Japan), Chugai Pharmaceutical (Tokyo, Japan), Taiho (Tokyo, Japan), Kyowa‐Kirin (Tokyo, Japan), Otsuka (Tokyo, Japan), Nihon‐Kayaku (Tokyo, Japan), Pfizer Japan (Tokyo, Japan), and Shionogi (Osaka, Japan) and received research funding from Chugai Pharmaceutical (Tokyo, Japan).
